# Protozoan co-infections and parasite influence on the efficacy of vaccines against bacterial and viral pathogens

**DOI:** 10.3389/fmicb.2022.1020029

**Published:** 2022-11-25

**Authors:** Lavoisier Akoolo, Sandra C. Rocha, Nikhat Parveen

**Affiliations:** ^1^Biorepository and Tissue Research Facility, University of Virginia School of Medicine, Charlottesville, VA, United States; ^2^Department of Microbiology, Biochemistry and Molecular Genetics, Rutgers New Jersey Medical School, Newark, NJ, United States

**Keywords:** protozoa, bacterial infection, co-infection, immune response, vaccine efficacy

## Abstract

A wide range of protozoan pathogens either transmitted by vectors (*Plasmodium*, *Babesia*, *Leishmania* and *Trypanosoma*), by contaminated food or water (*Entamoeba* and *Giardia*), or by sexual contact (*Trichomonas*) invade various organs in the body and cause prominent human diseases, such as malaria, babesiosis, leishmaniasis, trypanosomiasis, diarrhea, and trichomoniasis. Humans are frequently exposed to multiple pathogens simultaneously, or sequentially in the high-incidence regions to result in co-infections. Consequently, synergistic or antagonistic pathogenic effects could occur between microbes that also influences overall host responses and severity of diseases. The co-infecting organisms can also follow independent trajectory. In either case, co-infections change host and pathogen metabolic microenvironments, compromise the host immune status, and affect microbial pathogenicity to influence tissue colonization. Immunomodulation by protozoa often adversely affects cellular and humoral immune responses against co-infecting bacterial pathogens and promotes bacterial persistence, and result in more severe disease symptoms. Although co-infections by protozoa and viruses also occur in humans, extensive studies are not yet conducted probably because of limited animal model systems available that can be used for both groups of pathogens. Immunosuppressive effects of protozoan infections can also attenuate vaccines efficacy, weaken immunological memory development, and thus attenuate protection against co-infecting pathogens. Due to increasing occurrence of parasitic infections, roles of acute to chronic protozoan infection on immunological changes need extensive investigations to improve understanding of the mechanistic details of specific immune responses alteration. In fact, this phenomenon should be seriously considered as one cause of breakthrough infections after vaccination against both bacterial and viral pathogens, and for the emergence of drug-resistant bacterial strains. Such studies would facilitate development and implementation of effective vaccination and treatment regimens to prevent or significantly reduce breakthrough infections.

## Introduction

Co-infections of protozoa-bacteria-viruses are an emerging phenomenon due to overlapping epidemiological niches or shared transmission routes. Co-infections can adversely affect host immune responses, pathogenicity of microbes and success of chemotherapy and vaccinations. Co-infection of *Plasmodium* species with multiple bacterial species have been reported including with *Mycobacterium* (Chukwuanukwu et al., 2017), *Salmonella* (Cunnington et al., 2011), non-typhoid (NT) Salmonella (Takem et al., 2014), in addition to viruses such as HIV (Alemu et al., 2013), SARS-CoV-2 (Anyanwu, 2021), and hepatitis viruses (Helegbe et al., 2018). Such protozoan co-infections with bacterial and viral infections have high prevalence in same endemic regions especially in Sub-Saharan Africa. Malaria patients have been shown to be susceptible to other infections, influencing the pathogenesis and prognosis of the disease. Other interactions include *Babesia-Borrelia* (Dunn et al., 2014) as well as les common *Entamoeba* spp.-*Escherichia coli* have also been reported (Fernandez-Lopez et al., 2019). Alteration in the host, for instance, due to HIV infection that compromises host immune system can render humans more susceptible to co-infection by other opportunistic pathogens (Ayoade and Joel Chandranesan, 2022). Co-infecting pathogens may also have epidemiological implications and alter the mortality or morbidity due to diseases they cause (Anyanwu, 2021). Increase in DNA uptake and genetic recombination includes transfer of antimicrobial resistance genes between the co-infecting agents (Marti et al., 2017) resulting in emergence of multi-drug resistant pathogens. Apart from the effect on the pathogens, co-infections may also impact the efficacy of vaccines and success of chemotherapeutic agents.

Bacterial-protozoan co-infections are a commonly occurring phenomenon due to overlapping ecological niches and inadequate disease control infrastructures and thus, requires an all-inclusive approach to develop preventative vaccines and effective chemotherapeutic agents (Cox, 2001). Unexplained decline in efficacy of vaccines that are routinely used, and emergence of breakthrough infections is a concerning trend in disease endemic regions and poses a threat to control of infections around the world. The success of antimicrobials and vaccinations in diseases control depends heavily on a vibrant immune system. The immunodynamics of co-infections with protozoa need considered scrutiny during development of vaccines and antibiotics because the changes have the potential to lead to emergence of antigenic variations, breakthrough infections and antibiotic resistance (Wait et al., 2020).

The rise in immunocompromised individual numbers and accompanying vaccination failures has created a favorable microenvironment for emergence of more virulent pathogens (Laufer et al., 2007). This has further increased the urgency to investigate the causes of reduced effectiveness of vaccines against bacterial and viral pathogens to facilitate formulation of more inclusive and rational corrective measures. Inequitable resource distribution and marginalization of the developing world coupled with poor nutrition and disease control infrastructure complicates the control of global disease burden as more resistant strains emerge. These pathogens are then carried throughout the globe by traveling populations in now highly interconnected world (Walsh, 1989). This scenario creates an existential threat to disease control and a paradigm shift in approaches to vaccine and drug development. Therefore, it is critically important that acute to chronic protozoan infections and their presence during co-infections are considered seriously when more severe disease manifestations are noticed, or reduced vaccines effectiveness are observed.

**Figure 1:**
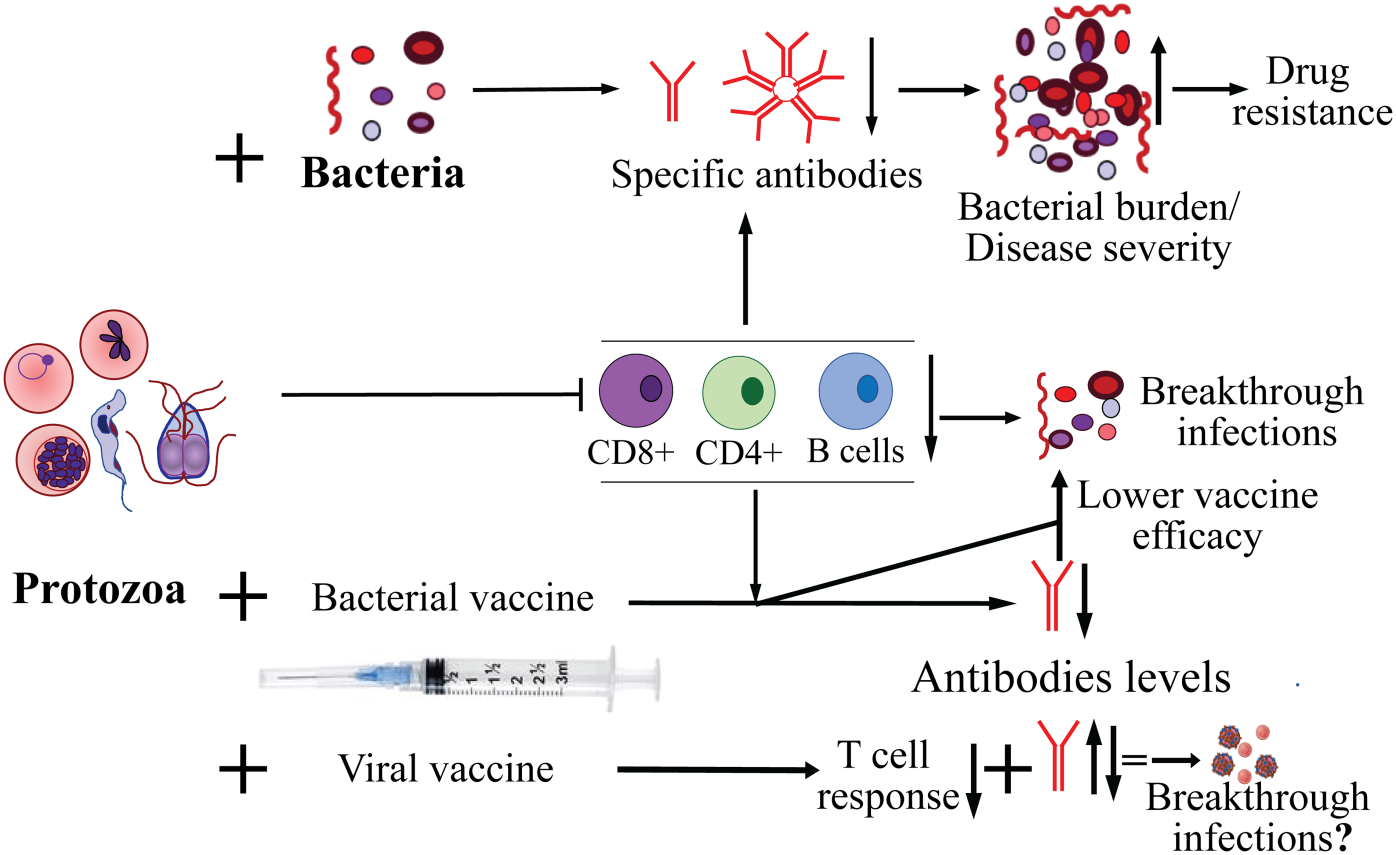
Effect of protozoan infection on adaptive immune response and co-infecting bacteria, and on effectiveness of bacterial and viral vaccines in protection from infection. The major pathogenic protozoa, such as *Plasmodium*, *Babesia*, *Trichomonas and Trypanosoma*, often cause suppression of adaptive immune response affecting both B and T cells. As a consequence, decrease in the specific antibody levels against co-infecting bacteria resulting in increase in burden of bacterial pathogens and exacerbation of severity of diseases they cause. Increased bacterial burden also enhances probability of emergence of resistance to antimicrobials. Diminished cellular and humoral immune response caused by acute to chronic infection by protozoa could also result in reduced efficacy/failure of vaccines against bacterial pathogens and may lead to breakthrough infections. Highly variable effects of protozoan infections with respect to antibody response to vaccines against different viral pathogens has been reported but their impact on breakthrough infections by the respective viruses remains to be investigated more thoroughly.

Based upon a comprehensive review of literature, we have summarized the impact of protozoan infections on pathogenesis of co-infecting bacterial and viral pathogens (Figure 1). We have also depicted the consequence of acute to chronic parasitic infection on emergence of drug-resistance in co-infecting pathogens and influence of protozoa on vaccines efficacy that could affect protection from infectious bacteria and viruses.

### The epidemiology of protozoa-bacteria co-infections

Protozoa-bacteria co-infections are an emerging healthcare problem especially in the developing world due to geographical overlap between different pathogens. For example, the overlapping existence of *Plasmodium* spp. and *Mycobacterium tuberculosis* that cause malaria and tuberculosis (TB), respectively particularly in countries with poor healthcare infrastructure creates a perfect setting for co-infections (Page et al., 2005). Co-infections involving gastrointestinal pathogens also commonly occur in low-income countries with poor water-sewer infrastructure and hygienic environments and thus, allow a common pathway of transmission (fecal-oral) as reported for *Campylobacter jejunum* and intestinal protozoa (Bronowski et al., 2014). Co-infections with sexually transmittable pathogens also occur frequently in women with abnormal vaginal bacteriome. Bacterial vaginosis (BV), a common syndrome when quantity and quality of vaginal microbiota is perturbed, often involves *Trichomonas vaginalis* infection (Onderdonk et al., 2016). *Clostridium perfringens* and *T*. *fetus* are also a frequent occurrence in bacterial-protozoan vaginosis. *Toxoplasma gondii* infections have also been observed together with *Clostridium perfringens* during endometritis (Alsammani et al., 2012). The presence of infected companion feline hosts with susceptible humans serves as the most ardent predisposing factor for *T*. *gondii* infection cycle. For respiratory and gastrointestinal co-infections, other common predisposing conditions in humans is underlying immunocompromised status, such as during HIV infection/AIDS or the presence of other enteric pathogens like entero-viruses.

Common vector and reservoir host(s) harboring multiple pathogens have been observed frequently. *Babesia* species, which are protozoans transmitted by *Ixodes scapularis* ticks, are hemoparasites with life cycle and pathology similar to *Plasmodium* spp. and trigger comparable impact on mammalian hosts immune responses and show several overlapping diseases manifestations (Djokic et al., 2021). Lyme disease causing spirochetal bacteria belonging to *Borrelia burgdorferi sensu* lato are also transmitted to different hosts by ticks (Stewart and Bloom, 2020). Increasing rate of co-infections by these two pathogens have been reported in the endemic regions of the United States (Swanson et al., 2006; Diuk-Wasser et al., 2016; Wormser et al., 2019) and Europe (Olsthoorn et al., 2021). Thus, in addition to *B*. *burgdorferi*-*Babesia* co-infections, simultaneous *Babesia*-*Anaplasma phagocytophilum* infections are also reported because tick vector and white footed mouse, *Peromyscus leucopus* (and other reservoir hosts) harbor several pathogens together. While some protozoa have established a mutual relationship with bacteria in which each microbe benefits from the infected host, other examples may involve cases where the pathogen modulates the immune status to benefit itself at the expense of the host (Schmid-Hempel, 2009).

### Protozoan infection and adaptive immune response

Following infection, protozoan parasites may either induce antibody, cell mediated immunity or stimulate both types of immune responses. These changes depend on the type of infection, localization of pathogen in the body, and the development stage of the organism. Various protozoan pathogens colonize different organs of the body, such as gastrointestinal tract (*Amoeba*), blood stream (*Trypanosoma*), within erythrocytes (*Plasmodium* and *Babesia*) and inside the macrophages (*Leishmania* spp., and *T*. *gondii)*. Extracellular parasites generally induce and can often be controlled by antibody mediated killing by opsonophagocytosis while intracellular protozoan pathogens control requires cell mediated immune responses (Bretscher, 1992). *Leishmania* spp. are obligate intracellular protozoan, such that defense mechanisms against this parasite depend upon CD4+ T-lymphocytes stimulation that could also activate macrophages and induce Th1 cytokines production (Gupta et al., 2013). Some parasites can induce both humoral and cell mediated immunity depending on the developmental stage of the pathogen, for, e.g., in *Plasmodium* spp. (Beck et al., 1995). In addition, they may also deploy immune evasion mechanisms for survival in the hosts, such as escape from antibodies by changing their surface antigenic coats, i.e., antigen variation observed in *Trypanosoma brucei* (Márquez-Contreras, 2018). *Trypanosoma brucei gambiense* induces humoral immune response because of its extra-cellular existence; however, antigenic variation of the parasites is hallmark of its long-term persistence in the host because it enables the protozoan to evade the immune system-mediated elimination in many cases (Márquez-Contreras, 2018).

### The pathogenesis of protozoan-bacterial co-infections

Protozoan-bacterial co-infections affects hosts in many ways. They can either lead to an antagonistic/deleterious or synergistic/advantageous pathogenic effect on the infected hosts. Antagonisms may be caused by resource competition or stimulation/suppression of innate or adaptive immune response that could negatively affect the co-infecting pathogen (Rigaud et al., 2010). During synergistic interactions, pathogens could suppress immune response of hosts resulting in high rates of replication of one or both pathogens. For example, protozoan infections can lead to apoptotic clearance of cells of the immune system creating a favorable environment for their own multiplication and potentially for proliferation of the co-infecting bacteria (Gigley et al., 2012). In addition, co-infecting pathogens can also modulate the gene expression in each other. Furthermore, synergism may also involve direct or indirect resources sharing, such that one microbe assists the co-infecting pathogen with regards to acquisition of nutrients (Birger et al., 2015). In this case, one pathogen may create favorable environment for colonization and growth of another pathogen, for, e.g., the presence of bacterial biofilm may promote proliferation of protozoa to stimulate synergism (Denoncourt et al., 2014). Alternatively, each pathogen takes on independent mechanisms of existence with minimal effect on diseases they cause. Several examples listed below demonstrate different effects of protozoan-bacterial co-infections on each other and on host(s).

#### *Plasmodium* and *mycobacterium tuberculosis*

Two major diseases, TB and malaria commonly occur together in patients due to overlapping geographical regions of infection for the two causative organisms (Baluku et al., 2019). Due to some similar initial subjective manifestations: flu-like illness, chills, fever, and fatigue, diagnostic approaches can miss some infections that leads to delayed therapeutic intervention. Thus, the lack of treatment for asymptomatic parasitic infection can apply selection pressure on TB by facilitating the emergence of drug-resistant strains (Murray et al., 2014). Interestingly, *P*. *falciparum* and *P*. *vivax* infections showed significant reduction in B and T (both CD4+ and CD8+) cells in patients (Kassa et al., 2006). *M*. *tuberculosis* infection has also been demonstrated to modulate the immune responses and confer immunological protection against severe malaria while weakened responses occur against bacteria. As a result, *Plasmodium-M*. *tuberculosis* co-infected patients show reduced/mild symptoms of malaria and a more severe symptoms of tuberculosis (Chukwuanukwu et al., 2017). In a murine model of infection, *M*. *tuberculosis*-induced potentiation of type 1 immune responses has been associated with protection against lethal malaria, which also appears to be related to induced production of IFN-γ and TNF-α in C57/BL6 mice (Page et al., 2005). Furthermore, mice sequentially infected with *M*. *tuberculosis* followed by *P*. *yoelii* were less capable in containing bacterial growth in lungs, spleen, and liver and resulted in increased mortality of mice (Scott et al., 2004). Also, increased *M*. *tuberculosis* burden were observed in lungs of mice co-infected with *P*. *berghei* (Mueller et al., 2014) or *P*. *yoelii* (Blank et al., 2016). While this co-existence exacerbates disease by *M*. *tuberculosis*, it is antagonistic to *Plasmodium*-induced illness. Interestingly, heat shock protein 70 (HSP70) from *M*. *tuberculosis* has been associated with the induction of a strong humoral and cellular response directed against *P*. *falciparum* (Page et al., 2005). During malaria, a marked increase in the production of the anti-inflammatory cytokines IL-10 and IL-4 (Chukwuanukwu et al., 2017) occurs and is thought to exacerbate TB pathology by reducing the protective Th1 bias and tilting immunity towards Th2 response. Additionally, co-infection with the non-lethal *P*. *yoelii* also resulted in more severe tuberculosis pathology with increased immune cells infiltration, and increased pro-and anti-inflammatory mediators, mainly IFN-γ, TNF-α, IL-6, IL-10, and IL-17. Moreover, higher TNF-α levels positively correlated with increased *M*. *tuberculosis* burden in lungs of co-infected mice (Blank et al., 2016).

#### *Plasmodium* and non-typhoid salmonella

Co-infections between Non-Typhoid Salmonella (NTS) and highly pathogenic *Plasmodium* species have also been reported (Mtove et al., 2011). *Plasmodium* infection causes extensive hemolysis and release of cellular heme, which can be toxic for organisms. The protozoan converts it to hemozoin, a novel non-DNA ligand for Toll-like receptor (TLR)9, which can be captured by cells of the reticuloendothelial system (RES) and can activate innate immune responses (Simoes et al., 2015). In response, mammalian host produces heme oxygenase-1 to degrade the heme and mitigate malaria pathology by limiting production of reactive oxygen species (ROS; Gozzelino et al., 2010); however, reduced ROS has counterproductive effect due to decrease in beneficial granulocyte oxidative burst function in clearing infections, and thus allows multiplication of co-infecting *Salmonella* in neutrophils (Cunnington et al., 2012; Harding et al., 2020). Activation of TLR9 also results in the production of pro-inflammatory cytokines, certain chemokines, and causes up-regulation of costimulatory molecules (Coban et al., 2005).

#### *Plasmodium* and other bacterial infections

Bacterial infections offer major complication during *Plasmodium* co-infections. During rodent *P*. *yoelii* infection, host immunity is impaired against diverse bacteria, including *Streptococcus pneumoniae* due to effects on innate immune response (Harding et al., 2020). A parasite-specific factor (haemozoin and bound bioactive molecules) directly contributes to *Plasmodium*-induced suppression of innate immune response against bacteria. *P*. *yoelii* infections also suppresses immune responses against *Listeria monocytogenes* by causing increased apoptosis of *Listeria*-specific T cells resulting in slower induction of cellular immune responses. Interaction between different strains of *S*. *pneumoniae* and the rodent malaria parasite *P*. *chabaudi* have also been shown to promote an antagonistic sequelae (Moens et al., 2012; Fairlie-Clarke et al., 2013). Studies have also shown that complement components, C1q and C3, interact with *P*. *falciparum* infected RBCs to initiate the complement cascade that leads to complement depletion (Nyakoe et al., 2009), which has been attributed to increased burden of *S*. *pneumoniae* during *Plasmodium*-*S*. *pneumoniae* co-infections (Harding et al., 2020).

#### *Babesia* spp. and *Borrelia burgdorferi*

*Babesia-B*. *burgdorferi* co-infections are a common occurrence due to shared reservoir host(s) and vector (Benach et al., 1985; Krause et al., 1996; Wormser et al., 2019). Studies in mice have demonstrated that when infected with *B*. *burgdorferi*, mice have been found to exhibit features similar to those of human Lyme disease (Barthold et al., 2010). During experimental co-infection of susceptible mice, *B*. *microti* infection causes splenomegaly and splenic dysfunction that results in a reduction in the levels of functional B and T cells. As a result, the production of specific antibodies against both pathogens are reduced causing poor control of *B*. *burgdorferi* infection (Djokic et al., 2019). Diminished adaptive immunity then exacerbates Lyme disease severity that is indicated by both; increased burden of *B*. *burgdorferi* in different organs of co-infected mice and more severe Lyme arthritis compared to those in mice infected with Lyme spirochetes alone (Parveen and Bhanot, 2019). These results agree with previous investigation showing that *B*. *burgdorferi* infection increased Lyme arthritis severity in co-infected Balb/c mice compared to singly infected mice (Moro et al., 2002). Limited human epidemiological studies have been conducted to determine outcomes of *Babesia*-*Borrelia* co-infection [reviewed (Knapp and Rice, 2015)] but overall, more diverse and persistent manifestations associated with *B*. *burgdorferi* infection were observed in co-infected patients (Krause et al., 1996). Thus, limited clinical studies have shown some overlapping features with those observed in mice; however, more thorough investigations are needed to fully determine the impact of co-infections on each disease severity.

#### *Trypanosoma brucei* and *Brucella*

During infection with *T*. *brucei*, phagocytosis of the protozoan has been found to be associated with an extensive production of cytokines. Cytokines IFN-γ and TNF-α were shown to be involved in exacerbation of anemia as mice lacking the respective genes exhibited protection from anemia, while anti-inflammatory cytokine, IL-10 counteracted the effects of *Trypanosoma*-induced anemia (Tabel et al., 2008; Musaya et al., 2015). Mice infected with *T*. *brucei* exhibit the characteristic parasitemia waves concurrently with the host expression of elevated levels of IFN-γ. Trypanosomes overcome host innate immune response and then cause significant immunosuppression allowing proliferation of this pathogen. The induction of pro-inflammatory IFN-γ by host in response to *T*. *brucei* infection has been shown to reduce splenic bacteria burdens in mice infected with either *Brucella melitensis*, *B*. *abortus*, or *B*. *suis* (Machelart et al., 2017). *T*. *brucei-Brucella* co-infection is therefore antagonistic for *Brucella*. In other cases, induction of IFN-γ is not sufficient to control selected bacterial infections, for, e.g., *M*. *tuberculosis* (Vilaplana et al., 2014; Musaya et al., 2015).

#### *Trichomonas vaginalis*, *Mycoplasma hominis*, *Atopobium* spp. and *Gardnerella* spp.

Trichomoniasis, a prevalent sexually transmitted infection (STI) is caused by the protozoan parasite *T*. *vaginalis*, which can establish a symbiotic relationship with *M*. *hominis*, a species implicated in bacterial vaginosis (Rappelli et al., 1998). *M*. *hominis* synergistically upregulates human monocytes pro-inflammatory response to *T*. *vaginalis* resulting in enhanced inflammation during trichomoniasis (Fiori et al., 2013). Due to influence of *Trichomonas* on change in vaginal pH, increase in infections in the urogenital tract also include other bacterial vaginosis associated bacteria. For example, co-existence of *Atopobium* and *Gardnerella* have also been found to cause synergistic enhancement of *T*. *vaginalis* induced production of chemokines (Onderdonk et al., 2016).

#### Protozoa-bacteria co-infection of gastrointestinal tract

Bacterial-protozoan co-infections also affect the gastrointestinal system with significant implications to the ensuing pathology. *E*. *histolytica* is a pathogenic protozoan related to intestinal and extraintestinal infections. In the large intestine, it co-exists with many resident microbiotas and results in asymptomatic infection or diarrhea (Stanley, 2003). The protozoan must compete with indigenous bacteria and may breach the mucus barrier. After binding to host cells, protozoan induces cell death, which causes amebic colitis and facilitates dissemination into extraintestinal organs. The interaction between *E*. *histolytica* and *E*. *coli* O55 causes substantial changes in their genes’ expression (Fernandez-Lopez et al., 2019). *E*. *coli* offers nutritional support for amoebic growth and helps the parasite to boost defenses against H_2_O_2_ induced oxidative stress to facilitate establishment of parasitic persistence in intestinal mucosa. As an example, oxaloacetate produced by *E*. *coli* protects *E*. *histolytica* against H_2_O_2_ induced oxidative stress while epithelial monolayers exposed to enteropathogenic bacteria are more susceptible to additional damage *by E*. *histolytica*. Phagocytosis of pathogenic/non-pathogenic bacteria promoted by amoebae further increased epithelial cells layer damage and exacerbated colitis severity (Galvan-Moroyoqui et al., 2008).

Murine studies have shown that *Giardia intestinalis*-enteroaggregative *E*. *coli* (EAEC) co-infection promotes bacterial growth impairment, microbiota-dependent delayed parasite clearance, microbial metabolic perturbations in the gut, and an alteration of localized host immune responses against EAEC (Bartelt et al., 2017). In contrast, *G*. *muris* reduces the symptoms of *Citrobacter rodentium*-induced colitis, by enhancing the production of mucosal antimicrobial peptides such as mouse β-defensin 3 and Trefoil factor 3 (Manko et al., 2017).

*Helicobacter pylori* (*H*. *pylori*) and *Cryptosporidium* spp. are well-known for their high prevalence in immunocompromised pediatric patients worldwide especially in developing countries (Ibrahim et al., 2019). *H*. *pylori* may support the colonization by *Cryptosporidium* spp. and vice versa. The interaction between *H*. *pylori* and intestinal parasites may have serious health consequences because Cryptosporidiosis results in increased intestinal permeability while *H*. *pylori* causes atrophic changes in the stomach. Together they may have a serious impact on tissue integrity and the balance of gut microbiome. Further investigation is warranted to unravel how this interaction affects the gut microbiome.

### The epidemiology of protozoan parasite-viral co-infections

The interactions between viruses-protozoan co-infections and their complexities remain unexplored. These pathogens can reciprocally alter their epidemiology and/or host response to vaccines and therapies (Karp and Auwaerter, 2007). Virus-protozoan co-infections such as those caused by HIV and *Plasmodium* have been documented particularly in sub-Saharan Africa. Co-existence of these pathogens represents an emerging healthcare problem that has been causing significant morbidity and mortality, with more than 2 million deaths occurring annually (World Health Organization, 2017). Several other studies demonstrated that people living with HIV have more frequent and severe malaria manifestations (World Health Organization, 2015). HIV infected individuals are also at higher risk of exposure to leishmaniasis (Okwor and Uzonna, 2013; Diro et al., 2014) and have more efficient *T*. *gondii* infection with increased risks of deaths (Pott Jr. and Castelo, 2013). In addition, people with HIV have up to 21% more seroprevalences against amebiasis (Hung et al., 2012) and have significantly higher rates of trichomoniasis than HIV-negative individuals (36.4% vs. 21.3%) (Davis et al., 2016).

Other *Plasmodium*-viral co-infections are also frequently observed in sub-Saharan Africa. A study from Anastos et al., 2010 in Rwanda (East Africa) showed an association between *Plasmodium* infection and increases risk of cervical precancer in Human Papilloma Virus (HPV) infected patients (Anastos et al., 2010). In a recent study from Nigeria (West Africa), authors reported that *Plasmodium* infection coexists with Measles virus in 32.5% febrile children analyzed and they were under risk of serious consequences or even death (Aminu et al., 2021). Nigeria also has high rates of malaria-influenza co-existence among people refusing flu vaccinations. Influenza A and B were found in 54% of unvaccinated pregnant women having *Plasmodium* parasitemia (Anjorin and Nwammadu, 2020). *Plasmodium* spp. and hepatitis B Virus (HBV) infections are prone to co-exist in individuals living in the same regions. In a systematic review and meta-analysis conducted, 22 studies were analyzed and showed that overall co-infection prevalence between *Plasmodium* spp. and HBV is 6% worldwide with the highest prevalence rate (10%) in Gambia (Kotepui and Kotepui, 2020). Strong positive association was also found between seropositivity for *Plasmodium* and Ebola virus in residents from Gabon, Central Africa with co-infection prevalence of 10.2% (Abbate et al., 2020).

*Cryptosporidium* is one of the most important parasitic diarrheal agents affecting children in the developing countries (Tamomh et al., 2021). *C*. *hominis*, *C*. *parvum* and *C*. *meleagridis* have been implicated in diarrhoea. This protozoan has also emerged as a global opportunistic threat causing severe diarrhea (Ahmadpour et al., 2020). *Cryptosporidium* infection is common among HIV/AIDS patients (prevalence of 8,69%) worsening the protozoan infection associated symptoms causing severe diarrhea and eventually death because of low CD4+ T-cells counts (Wang et al., 2013; Fregonesi et al., 2015; Hailu et al., 2015; Yang et al., 2017; Wang et al., 2018). As a consequence of such co-infection and severe disease (Fregonesi et al., 2015; Alemu et al., 2018) parasitemia as high as 90% was observed (Ojurongbe et al., 2011).

Due to more recent emergence of infection by severe acute respiratory syndrome coronavirus 2 (SAR-CoV2) in humans, co-infections with different protozoan are not fully explored yet. Moreover, most of the available reports about SARS-CoV2 co-infections describe concomitant bacteria, fungus and other viral infections, especially associated with respiratory infections and pneumonia. In depth studies have not been conducted for SARS-CoV2 and protozoan co-infections; however a few reports are available showing co-infections occur with *Toxoplasma* (Montazeri et al., 2022), *Plasmodium* (Raham, 2021; Boonyarangka et al., 2022), *Babesia* (Jacobs and Siddon, 2021), *Leishmania* (Pikoulas et al., 2022) and *Trypanosoma* (Alberca et al., 2020). In the first year of the COVID-19 pandemic, worldwide malaria cases increased from 227 million in 2019 to 241 million in 2020 (World Health Organization, 2021) and a high rate of latent *T*. *gondii* infection was also found among COVID-19 patients with severe manifestations reported in the Middle East region (Montazeri et al., 2022). Therefore, more research is needed to fully understand the impact of SARS-CoV-2 infections on co-infecting protozoa, and vice versa.

### The pathogenesis of protozoan-viral co-infections

#### *Plasmodium* and HIV

Due to the geographical overlap between *Plasmodium* and HIV, both pathogens can often infect humans with synergistic and adverse impact (Alemu et al., 2013). HIV infection increases the *Plasmodium* burden in patients, facilitating the increase in protozoan transmission. Conversely, infection with *Plasmodium* results in increase in number and activation state of CD4 + T cells, creating an ideal environment for HIV replication, increasing viremia. Other mechanisms of *Plasmodium*-infection induced HIV replication include the secretion of TNF-α that can act directly stimulate HIV replication (Ayouba et al., 2008). Additionally, infection with *Plasmodium* causes pro-inflammatory (T helper 1- type) immune response with activation of CD4+, CD4 + 5RO + T cells. These T cells are preferred target for HIV replication (Spina et al., 1997). CD14+ macrophages activated during acute malaria are also a source of migratory reservoirs of HIV-1 facilitating dissemination of virus to lymphocytes during cell–cell interactions promoting disease dissemination (Pantaleo and Koup, 2004). *P*. *falciparum* has also been shown to stimulate HIV-1 replication through the production of cytokines (IL-6 and TNF-α) that activate lymphocytes (Inion et al., 2003). Other studies have shown that exposure to soluble *Plasmodium* antigens and hemozoin induced HIV replication or reactivation *via* CD4 T-cell stimulation together with the production of pro-inflammatory cytokines, for, e.g., IL-1β, IL-6, and TNF-α (Froebel et al., 2004).

#### Co-infections with SARS-CoV2

It has been hypothesized that malaria may reduce the COVID-19 severity in endemic regions of sub-Saharan Africa (Gutman et al., 2020; Ssebambulidde et al., 2020; Osei et al., 2022). An inverse correlation between the incidence of COVID-19 and malaria with less probability of COVID-19 cases was found in malaria-endemic countries (Ssebambulidde et al., 2020). One possible explanation for this phenomenon is that malaria patients generate anti-GPI antibodies which eventually identify SARS-CoV-2 glycoproteins developing a protective response against COVID-19 improving the disease prognostic (Hussein et al., 2020). Conflicting results from a Malian longitudinal cohort study showed no association between malaria and COVID-19 seroconversion or effect on the symptoms reported for COVID-19 (Woodford et al., 2022). The identification of immunomodulatory effects provoked by malaria and helminth infections could lead us to better understanding of the factors involved in improvement of vaccine efficacy. It still remains unclear how efficacy of COVID-19 vaccines is affected by these parasites.

There are several reports showing association between Neglected Infectious Diseases (NTDs) and SARS-CoV2, in terms of how they affect the severity of COVID-19 clinical outcomes, vice versa and the development of trained immunity as occurs for helminth infections and malaria (Ssebambulidde et al., 2020; Anyanwu, 2021; Gluchowska et al., 2021; Wilairatana et al., 2021; Achan et al., 2022; Hussein et al., 2022). Our review of literature indicate that the immunomodulatory effects of COVID-19 and parasitic co-infections brought insights not by direct investigations but based upon lessons learned from other co-infections systems (Fonte et al., 2020; Gluchowska et al., 2021; Akelew et al., 2022; Woodford et al., 2022). Briefly, helminth co-infection was suggested to cause immunomodulation in COVID-19 patients to result in reduction of disease severity (Bradbury et al., 2020). This immunomodulation could be due parasite specific innate response and Th2 immune response with CD4+ T cells, eosinophils, and production of IL-4, IL-5, and IL-10, thereby reducing hyperinflammation in patients with severe COVID-19 (Rodriguez, 2020; Akelew et al., 2022). Reinforcing these observations, a recent study showed that patients co-infected with SARS-CoV2 and helminths had less severe COVID-19 due to reduced hyper-inflammation response (Wolday et al., 2021). In fact, an inverse correlation between COVID-19 existence and severity was observed in countries endemic for soil-transmitted helminths (Ssebambulidde et al., 2020).

#### *Plasmodium* and SARS-CoV-2

*Plasmodium* and SARS-CoV2 co-infections have been reported to occur across the endemic and non-endemic regions (Junaedi et al., 2020). Despite the rising incidence of COVID-19 disease in the world, an unremarkably lower prevalence has been observed in malaria endemic regions (Osei et al., 2022) suggesting that *Plasmodium* presence may offer some protection against SARS-CoV-2 infection. SARS-CoV-2 uses the angiotensin-converting enzyme 2 (ACE2) receptor to enter the host cells. However, a D-allele variant of ACEI/D polymorph has been described in a mild form of malaria. This ACE I/D polymorphism occurs in intron 16 reduces ACE2 expression. Reduced expression of ACE2 receptor in populations with this polymorphism may play a protective role against severe COVID-19. An increase of its substrate Ang II in plasma of these individuals have been demonstrated in people with African genetic background (Delanghe et al., 2020).

At the height of the COVID-19 outbreaks, relatively low prevalence rates were observed in areas known to have high malaria endemicity, prompting some interest on the role of malaria immunity in protecting COVID-19 infections (Vilaplana et al., 2014). Infections with *Plasmodium* induces both innate and adaptive immunity. Recent studies have shown that in addition to inducing adaptive B and T cells memory response, innate immune response to *Plasmodium* infection may also induce memory, a phenomenon known as trained immunity, which is capable of mounting a faster and more robust recall response and may provide cross protections against unrelated pathogens (Netea et al., 2011, 2020). Cross protection induced by trained immunity is a widely acknowledged phenomena and has been demonstrated by BCG vaccinations against *M*. *tuberculosis* because it provides cross protection against unrelated pathogens (Sohrabi et al., 2020). The major factors involved in innate immune response to malaria include natural killer (NK) cells, monocytes, macrophages, and pro- and anti-inflammatory cytokines (Hansen et al., 2007; Doolan et al., 2009). These responses can develop nonspecific trained immunity that can be effective against other pathogens like SARS-CoV-2, producing a faster and more effective protective response (Raham, 2021). Trained immunity against *Plasmodium* could also produce tolerance that tapers down the inflammatory response from innate immune cells, such as monocytes (Boutlis et al., 2006). Tolerance has the beneficial effect of reducing the harmful effect of excessive infection and disease (Nahrendorf et al., 2021), and cross-protection could occur by reduction of the inflammatory progression to unrelated disease, including SARS-CoV2. Such a response may explain the reduced COVID-19 severity in malaria community (Guha et al., 2020). Further research is needed to examine these concepts as relevant to COVID-19 in malaria endemic areas. For example, the innate immune factors (NK cells, Type 1 IFN, IgG) need to be evaluated in COVID-19 asymptomatic and symptomatic patients in malaria endemic areas.

#### *Plasmodium* and other viral co-infections

Co-infections by *Plasmodium* and hepatitis B and C (HBV and HCV) viruses often occur due to shared needles and blood transfusions etc. (World Health Statistics, 2022). A high incidence rate of malaria and hepatitis infection in the sub-Saharan population has been reported (Helegbe et al., 2018; Sevede et al., 2019). Co-infections with *Plasmodium* and hepatitis often results in changes in burdens of both pathogens such that individuals co-infected with *Plasmodium* spp. and HBV display lower parasitemia and higher viremia (Andrade et al., 2011). Both of these pathogens also have an antagonistic effect on anemia, while *P*. *falciparum* causes hemolytic anemia, HBV increases hemoglobin levels by releasing erythropoietin from regenerating hepatic tissues (Simon et al., 1982; Klassen and Spivak, 1990; Ifudu and Fowler, 2001). Conversely, during chronic HBV infection, cytokines released in response to *P*. *falciparum* infection could further activate the apoptosis of HBV-infected hepatocytes, and exacerbated liver damage as evidenced by the increase in pro-inflammatory cytokines, TNF-α, IL-1β, and IL-6 that are increased during *Plasmodium* and chronic HBV infection and reduction in anti-inflammatory cytokines IL-10 and IL-4 in pregnant women (Brown et al., 1992; Azizieh et al., 2018).

Historically, Ebola outbreaks have occurred in Western Africa, and it overlaps with malaria prevalence. Studies have shown that *P*. *falciparum* infection prior to infection with Ebola virus could induce an antiviral activity and a protective role against Ebola. Acute *Plasmodium* infection has been shown to promote IFN-γ-dependent resistance to Ebola virus infection (Rogers et al., 2020).

Outbreaks of measles have been reported in malaria endemic areas of sub-Saharan Africa generating some interest on the impact of one pathogen over the other albeit studies to-date are limited. One study has shown significantly lower parasitic prevalence and mean densities of malaria parasites were found in children up to 9 years of age who had measles or influenza than in asymptomatic control children (Rooth and Bjorkman, 1992).

#### *Cryptosporidium* and HIV

Pathogens belonging genus *Cryptosporidium* are transmitted by fecal-oral route causing gastrointestinal infection in various vertebrate species, including humans (Xiao et al., 2004; Wang et al., 2011) and has been associated to chronic to life-threatening diarrhea in immunocompromised individuals (Conner et al., 2019). Both innate and adaptive immune responses play a role in protection from cryptosporidiosis and resolution of infection; however, cell-mediated immunity is crucial for clearance of cryptosporidiosis (Borad and Ward, 2010). HIV/AIDS patients with lower CD4 counts are more susceptible to cryptosporidiosis and have greater severity of disease (Hunter and Nichols, 2002). In HIV/AIDS patients with active cryptosporidiosis, infected epithelial cells express high levels of the chemokine, CXCL10, and expression levels correlate with the parasite burden (Wang et al., 2007). Since CXCL10 increases the rate of HIV replication *in vitro*, elevated CXCL10 in cryptosporidiosis may contribute to enhanced destruction of CD4+ T cells due to HIV infection (Ahmadpour et al., 2020). Humoral immune responses have also been reported to play an important role in protection against Cryptosporidiosis since studies have suggested that that antibody responses to specific antigens were associated with protection from diarrhea in *Cryptosporidium*-infected HIV/AIDS patients (Ahmadpour et al., 2020). Specific serum IgG, IgM, and IgA production were evaluated in *Cryptosporidium*-HIV co-infection showing no difference among patients with or without diarrhea (Kaushik et al., 2009). The occurrence of diarrhea in HIV-positive individuals was not always observed during *Cryptosporidium* co-infections probably because antiretroviral therapy improved the immune system functionality (Irisarri-Gutierrez et al., 2017). Conversely, *Cryptosporidium* has been shown to stimulate periductal inflammation in the biliary tree, induces biliary epithelial cell apoptosis, and thus could contribute to the pathogenesis of AIDS-cholangiopathy (Chen and LaRusso, 2002).

#### *Toxoplasma* and HIV

*Toxoplasma gondii*, a coccidian protozoan obligate intracellular parasite, is the causative agent of toxoplasmosis which affects approximately 30% population worldwide. *T*. *gondii* infection results in malformation and life-threatening disease in developing fetuses and is one of the most prevalent causative agents of opportunistic infections in HIV/AIDS patients causing central nervous system toxoplasmosis (Montoya and Liesenfeld, 2004; Ayoade and Joel Chandranesan, 2022). According to a systematic review, toxoplasmosis co-infection prevalence in HIV patients varies greatly among different countries showing the highest numbers in Thailand (53.7%), North Sudan (75.0), Ethiopia (87.4%), Brazil (80.0%) and Iran (96.3%). These authors also observed that high prevalence of *T*. *gondii*-HIV co-infections in low-income countries (Wang et al., 2017). It has been shown that *T*. *gondii*-HIV co-infections lead to toxoplasmosis severity and increase mortality in patients who developed AIDS and were not properly treated (Da Cunha et al., 1994; Pott and Castelo, 2013; Agrawal et al., 2014). During HIV infection, depletion of CD4 cells, decreased production of cytokines and IFNγ, and impaired cytotoxic T-lymphocyte activity result in reactivation of latent *Toxoplasma* infection (Basavaraju, 2016), and is associated with persistence of IgG antibodies against *T gondii* (Robert-Gangneux and Darde, 2012). Additionally, low CD4 T lymphocyte count was associated with high frequency of encephalitis caused by toxoplasmosis in patients who developed AIDS (Lejeune et al., 2011). On the other hand, *T*. *gondii* co-infection also alters the immune response, clinical manifestation and transmission of the HIV infection (Welker et al., 1993; Bala et al., 1994). In individuals singly infected with HIV, for example, both numbers of plasmacytoid dendritic cells and IFN-α production are impaired (Feldman et al., 2001), which can be exacerbated during opportunistic *Toxoplasma* co-infection. Additionally, the exposure to *T*. *gondii* has been shown to potentiate CD4 positive T-cells and possibly monocytes to be more permissive for HIV replication (Subauste et al., 2004), suggesting that HIV/*T*. *gondii* co-infected individuals potentially exhibit more severe diseases.

#### Other protozoan-viral co-infections

Preliminary studies have shown that toxoplasmosis is a risk factor for acquiring SARS-CoV-2 infection and severe manifestations of COVID-19 (Fiori et al., 2013). *T*. *gondii* induces the shedding of mitochondrial outer membrane to promote its own growth. Intriguingly, the hijacking of host mitochondria has been shown to play a critical role in the pathogenesis of COVID-19 (Lo et al., 2014).

The relationship between HIV-1 infection and amoebiasis (Lowther et al., 2000; Hung et al., 2005, 2008) was demonstrated by the observation that HIV-infected men having sex with men (MSM) were at significantly higher risk of amebiasis than patients from other risk groups (Hung et al., 2008). One study has identified Amebic Liver Abscess (ALA) as the most common extraintestinal manifestation of invasive infection as an important condition in HIV-1-infected individuals and has been attributed to the ability of HIV-1 to suppress activity of regulatory T cells. This in turn suppresses *E*. *histolytica*-specific T-cell reactivity and cause increased susceptibility to invasive amebiasis in persons with early stage of HIV-1 infection (Hsieh et al., 2007).

*Trichomoniasis* is a highly prevalent STI among HIV-1-infected patients (Cu-Uvin et al., 2002). Previous investigation has demonstrated that *T*. *vaginalis* infection enhances HIV-1 transmission (Schwebke, 2005). Proposed mechanisms by which *T*. *vaginalis* infection may increase HIV-1 infection include: induction of inflammatory response in vaginal, exocervix, and urethral epithelia; disruption of mucosal barrier function; recruitment of CD4 lymphocytes and macrophages; development of microhemorrhages; degradation of secretory leukocyte protease inhibitors; and enhancement of susceptibility to bacterial vaginosis or other abnormal vaginal flora that all may increase the risk of HIV-1 acquisition (Sorvillo et al., 2001).

Rotavirus often contribute to the *Cryptosporidium* co- infection in farm animals and humans (Izzo et al., 2011; Mokomane et al., 2018; Praharaj et al., 2019). Differences in clinical manifestation between lambs infected with *Cryptosporidium* alone or together with rotavirus have been detected; however, some reports showed no differences during these two situations. More research is needed to reveal the influence of simultaneous occurrence of different pathogens, including *C*. *parvum*, which may either facilitate or antagonize concurrent infections. Some co-infections with *Cryptosporidium* species may not exert any response but needs to be investigated thoroughly.

### The impact of protozoan-bacterial co-infections on chemotherapeutic interventions

The emergence of co-infections could have a modulating effect the on the success or failure of chemotherapy by playing a role in the emergence of antibiotic resistant strains (Birger et al., 2015). Co-infecting pathogens are often misdiagnosed due to overlapping symptomatology, delay in treatment to allow excessive proliferation of the microbe(s) that can render the host immune response insufficient to clear infection. Increase burden can facilitate development of drug resistance in one or both pathogens (U.S. Department of Health and Human Services, 2018). Targeted treatment against one pathogen may also remove a competitor and could lead to active growth of the remaining pathogen increasing the probability of evolution of drug resistant variants as seen in malaria and TB (Colombatti et al., 2011). During synergistic infections, reduced immune system-mediated killing of pathogens may allow their replication, increasing the possibility of the emergence of *de novo* resistance.

Rifampicin is an antitubercular drug that also exhibits potent anti-malarial activity against *P*. *vivax* in humans (Pukrittayakamee et al., 1994). Continual usage of rifampicin against TB may have resulted in discontinuing its use against malaria parasite since the therapeutic doses for the two pathogens are different. Impaired immunological control of tuberculosis due to the presence of the co-infecting *Plasmodium* spp. may also increase the danger of a recrudescence of partially resistant pathogen populations after therapy has ended (Okeke, 2003). An indirect effect of antimicrobials to bioavailability of drugs, can be due to a physical hindrance provided by one pathogen, such as by forming a biofilm, leading to suboptimal drug concentration at the colonization site. The presence of co-infection may also increase the abundance of antibiotic-target pathogen, and thus aiding the focal infection.

Development of antimicrobials targeting common metabolic pathways of co-infecting pathogens is a promising field of research that could limit the establishment of multidrug resistance. For example, improvement in design of sulfonamide drugs to target the *de novo* folate synthesis pathway, which is used by both *Plasmodium* spp. and *M*. *tuberculosis* is an attractive idea for consideration in drug design. In fact, sulfonamide class of antibiotics, initially developed as antibacterial agents, have been central in the development of antifolate-based combinational drugs against malaria. Co-trimoxazole as an antibacterial prophylactic agent can also prevent the incidence of malaria. The long safety history of co-trimoxazole when used in pregnancy (to treat bacterial infections) and its antimalarial prophylactic properties have led to the evaluation of this combination to also prevent malaria during pregnancy.

### The impact of protozoan infections on the efficacy of bacterial/viral vaccines

Vaccines, like infections, involve participation of the innate and adaptive immune system which encompasses, phagocytosis, cytokine/chemokine secretion and activation of the antigen-specific adaptive immune response with subsequent immunological memory development. An effective adaptive immunity development involves activation of specific subsets of T lymphocytes, and stimulation of B lymphocytes to differentiate into antibody-secreting plasma cells followed by creation of protective immunological memory (Vetter et al., 2018). The critical starting point in the development of an effective vaccine requires identification of potent antigens that are appropriately presentable by professional antigen-presenting cells. Changes in the target antigens can impair development of an effective immune response. These changes may result from genetic variations arising as a consequence of co-infections resulting in failure of recognition by the adaptive immune response and occurrence of breakthrough infections (Bretsche et al., 2001). A previous meta-analysis study showed that parasitic infections such as those caused by helminths, protozoa and viruses at the time of vaccination were associated with worse immunological responses, tending to overcome infection less efficiently after post vaccination challenge. Multiple factors determine how parasitic infections impact the outcome of immunizations. These include: the type of parasite involved, immune response induced, vaccine formulation, route of administration, the target antigen, vaccine type (e.g., live attenuated, inactivated organism), study design and the timing of infection relative to vaccination (Wait et al., 2020).

The association between parasitic infections and impaired immune responses to vaccine antigens has been demonstrated for a diverse group of pathogens [(Wait et al., 2020) and Table 1]. For example, immune response induced by vaccines against *Haemophilus influenzae* and diphtheria (Malhotra et al., 2015), Bacille Calmette-Guerin (BCG) and tetanus toxoid (McGregor, 1962; Greenwood et al., 1972; Elliott et al., 2010; Alvarez-Larrotta et al., 2019), *S*. *typhi* (Williamson and Greenwood, 1978), acellular diphtheria-tetanus and pertussis vaccine (DTPa; Radwanska et al., 2008), HIV (Robinson et al., 2004), and potentially against SARS-CoV-2 too could be affected (Fonte et al., 2020; Gluchowska et al., 2021; Akelew et al., 2022). The impact of helminths on different vaccines outcomes has also been reported previously against pneumococcus (Apiwattanakul et al., 2014), BCG (Elias et al., 2008) and HIV-1C (Da’dara and Harn, 2010). Additionally, reduced vaccine efficacy has been associated with chronic helminth infections when compared to acute infections (Wait et al., 2020). In this review, we have focused on the impact of protozoan infections on the efficacy of vaccines against bacterial and viral infections.

**Table 1:** The most common protozoa co-infections and its effects on bacterial/viral vaccines efficacy.

Vaccine	Protozoan infection	Immunological response against vaccine	Effect on vaccine efficacy	References
BCG	*Plasmodium* spp.	B-cell depletion, loss of central memory CD4+ T cells	Vaccine efficacy not significantly altered	Tangie et al. (2022)
Different TB vaccines	*P*. *yoelii*	CD4 and CD8 T cells suppressed in mice at 2 weeks	*M*. *tuberculosis* CFU levels not affected	Parra et al. (2011)
Tetanus toxoid	*P*. *vivax* and *P*. *falciparum*	Lower expression of cytotoxic T lymphocyte antigen 4 and anti-toxoid IgG levels	Tetanus cases not evaluated	Alvarez-Larrotta et al. (2019)
Tetanus toxoid	*Plasmodium* spp.	Lower antibody response to tetanus toxoid in children with malaria	Tetanus cases not evaluated	McGregor (1962)
Tetanus toxoid and *Salmonella typhi*	*Plasmodium* spp.	Diminished antibody response to tetanus toxoid and *S*. *typhi* O antigen in children with acute malaria	Tetanus or *S*. *Typhi* cases not evaluated	Greenwood et al. (1972)
*S*. *typhi* and meningococcal vaccines	*Plasmodium* spp.	Antibody levels to both vaccines was significantly reduced when the vaccines were given on the first day of illness	Meningococcal disease or typhoid cases not evaluated	Williamson and Greenwood (1978)
*H*. *influenzae b* and diphtheria toxoid	*P*. *falciparum*	Impaired IgG antibody responses to *H*. *influenza* b and diphtheria among infants of mothers infected with malaria and/or helminths during pregnancy	*H*. *influenzae b* and diphtheria cases not evaluated	Malhotra et al. (2015)
*H*. *influenzae b*	*Plasmodium* spp.	11% of infected children with malaria did not have protective titers	*H*. *influenzae b* cases not evaluated	Usen et al. (2000)
*S*. *pneumoniae* and diphtheria CRM197 antigens	Malaria and some helminth diseases	Higher anti-vaccine antibody levels against *S*. *pneumoniae* and diphtheria CRM197 antigens during pregnancy	Throat infection, pneumonia and diphtheria cases not evaluated	McKittrick et al. (2019)
NTS serovars	*P*. *yoelii*	Marked reduction of *Salmonella*-specific CD4 and CD8 T cells immunity in mice	Reduced protection with 264-fold (liver), and 31-fold (spleen) increase in bacterial burden	Mooney et al. (2015)
*Brucella abortus* vaccine	*T*. *congolense*	Vaccinated cattle had depressed IgG1 and IgG2 levels by 80%, IgM by 90%.	Cattle not challenged with B. abortus	Rurangirwa et al. (1983)
*Bacillus anthracis* spore vaccine	*T*. *congolense*	The anti-anthrax antibody levels were severely depressed in infected goats	Goats not challenged with *B*. *anthracis*	Mwangi et al. (1990)
DTPa vaccine	*T*. *brucei*	Antibody response not determined against vaccine	Protection by DTPa vaccine eliminated	Radwanska et al. (2008)
HIV DNA vaccine	*L*. *major*	Significant reduction in IFN-γ-production by CD8+ T cells of Balb/c mice after *in vitro* stimulation with gag antigen	HIV vaccine efficacy not determined	Robinson et al. (2004)
HPV-16/18 vaccine	*Plasmodium* spp. + helminths	Association of malaria parasitemia in young girls with a higher level of anti-HPV-16/18 antibodies	HPV cases not evaluated	Brown et al. (1992)
Classical swine fever (CSF) vaccine	*T*. *evansi*	Antibody responses against CSF vaccine significantly reduced in pigs	More vaccinated animals had fever and leucopenia	Holland et al. (2003)
Foot-and-mouth disease vaccine	*T*. *congolense*	*T*. *congolense* infected cattle had antibody titers significantly depressed after secondary vaccination	After viral challenge, difference in protection was not significant in *T*. *congolense* infected group	Sharpe et al. (1982)
Influenza A Virus (IAV) Vaccine	*P*. *yoelii*	Plasma cell apoptosis and circulating BAFF increased, circulating IAV specific antibodies diminished	Higher virus load in challenged *P*. *yoelii* infected mice	Banga et al. (2015)
Tetanus, measles and hepatitis vaccines	*P*. *falciparum*	Declines in IgG specific for tetanus, measles and hepatitis B in 53, 19 and 33% of children, respectively	Incidence of infections not evaluated	Banga et al. (2015)
Measles vaccine	*P*. *falciparum*	Antibody titers were significantly higher in the vaccinated children positive for *P*. *falciparum*	Measles cases not evaluated	Smedman et al. (1986)
Rotavirus vaccine	*Cryptosporidium* spp. etc	Immune response not evaluated	Decrease of 11.3% in vaccine efficacy in co-infections. No statistically difference was found	Praharaj et al. (2019)
Rotavirus vaccine	*Cryptosporidium* spp., *Giardia* spp. etc	Immune response not evaluated	Protection by the vaccine was 59% in the rotavirus co-infection group and 51% in the rotavirus mono-infection subgroup, difference not statistically significant	Mokomane et al. (2018)

#### Protozoan parasites and vaccines against bacterial pathogens

Immunosuppression by protozoan pathogens could interfere with the immune response generated by vaccines, creating a negative correlation between parasitic infections and efficacy of vaccine in protection. Conversely, there is also evidence that vaccines may induce trained immunity and non-specific response against protozoa (Welsh and Selin, 2002; Selin et al., 2006; Agrawal, 2019) including those causing malaria and babesiosis (Clark et al., 1976, 1977; Garly et al., 2003; Walk et al., 2019). In fact, BCG vaccination, which can enhance non-specific protection to unrelated infections especially by activation of NK cells with non-specific memory by production of pro-inflammatory cytokines is provides an example (Kleinnijenhuis et al., 2014); however, parasitic infections often lead to lower antibody and IFN-γ levels, which represent a decrease in the quality of the humoral and cellular immune responses (Rowe et al., 2000).

Malaria is a highly prevalent disease in settings where poor responses to unrelated vaccines has been reported to occur (Natukunda, 2020). Several studies have shown that *Plasmodium* infection might impair vaccine induced protective immunity against other pathogens (Dietz et al., 1997; Cunnington and Riley, 2010). Diminished vaccines efficacy has been attributed to different factors, such as human and bacterial genotypes, exposure to environmental microbes, climate and geographical location and prevalence of co-infections (Tangie et al., 2022). A decreased protection against murine typhoid in *P*. *yoelii*-infected mice vaccinated with *S*. *typhimurium* antigens correlated with suppression of CD4 and CD8 T cell effector responses and increased anti-inflammatory IL-10 cytokine production (Mooney et al., 2015). *Plasmodium* spp. induced immunosuppression could be an important factor responsible for weak response to routine immunizations in malaria-endemic communities (McGregor, 1962; Gilles et al., 1983). For example, in a study in south coast of Kenya showed an impaired IgG antibody response to *H*. *influenza* b and diphtheria among infants of mothers infected with malaria and/or helminths during pregnancy compared to infants of uninfected mothers. The authors discussed that transplacental exposure of the fetus to parasite antigens in prenatal maternal malaria or helminth infections could lead an *in-utero* alteration of fetal immune responses affecting the response to vaccines (Malhotra et al., 2015). These observations were also confirmed in another study where Gambian children with malaria also had low antibodies titers to a *H*. *influenza* b conjugate vaccines than healthy controls, suggesting that the child cytokine profile at the time of antigen presentation likely modifies the immune response (Usen et al., 2000). Immune responses to both *Salmonella typhi* and meningococcal vaccines were also evaluated in Nigerian children infected with *Plasmodium*. Thus, *S*. *typhi* vaccination given on the first day of malaria infection had the immune responsiveness depressed, which rapidly recovered following malaria treatment while immune response to meningococcal vaccine was still impaired after a month of infection (Williamson and Greenwood, 1978). In contrast to all these findings, malaria and some helminth diseases during pregnancy were associated with minor, mostly enhancing effects on infant anti-vaccine antibody levels against *S*. *pneumoniae* and diphtheria CRM197 antigens (McKittrick et al., 2019).

Submicroscopic infection by *Plasmodium* spp. has been shown to be associated with a decrease in the levels of IgG against tetanus toxoid (McGregor, 1962; Greenwood et al., 1972; Alvarez-Larrotta et al., 2019), however, tetanus and diphtheria toxoids continue to offer protection in these individuals suggesting that functional immunity was sufficiently protective. Chronic protozoan infections can result in persistence of stimulating antigen that could lead to exhausted T cells with less robust effector functions, and alterations of the differentiation and sustenance of memory T cells (Costa-Madeira et al., 2022). Up-regulation and co-expression of multiple inhibitory receptors and failure to produce antigen-independent memory T cells are also observed in these cases (Schietinger and Greenberg, 2014).

Some studies showed that pre-existing *Plasmodium* infections did not affect the efficacy of vaccines against bacterial or viral pathogens, for instance, *P*. *yoelii* infection did not affect different formulations of TB vaccine ability to control pulmonary growth of an acute virulent *M*. *tuberculosis* infection (Parra et al., 2011). The immunological response to two doses of tetanus toxoid in groups of pregnant Kenyan women showed that the presence of *Plasmodium* parasitemia does not interfere with primary or secondary immune response during chemoprophylaxis against malaria (Dietz et al., 1997). Moreover, two doses of tetanus toxoid in 2-year-old Gambian children also showed that chloroquine or pyrimethamine chemoprophylaxis lead to more protective immune response when compared with children who were not treated for malaria (McGregor, 1962).

African trypanosomiasis caused by *T*. *brucei* infection inhibits protective immune responses against bacterium, *Bordetella pertussis* when immunized with a trivalent human vaccine, DTPa in mice (Radwanska et al., 2008). Other species of *Trypanosoma* can also prejudice the protective immune responses to bacterial or viral infections in animals. In an earlier study, cattle previously infected by *Trypanosoma congolense* when vaccinated against *Brucella abortus* had 80% reduction in IgG1 and IgG2 immunoglobulins (Rurangirwa et al., 1983). Furthermore, anti-anthrax antibody levels were severely depressed in *T*. *congolense* infected goats after immunization with *Bacillus anthracis* inactivated spore vaccine (Mwangi et al., 1990). Reduced antibody responses in infected pigs were also suggested to occur due to suppression of helper T cell caused by the concurrent *T*. *evansi* infection. In both mice and cattle, T cell proliferation was inhibited upon mitogenic stimulation and was mediated by macrophage-like suppressor cells, with reduction in IL-2 secretion together with impaired expression of the IL-2 receptor (Sileghem et al., 1989; Sileghem and Flynn, 1992). *T*. *brucei* infection also results in a rapid loss of B cells by apoptosis, reducing humoral immunity that further prevents the development of protective memory responses and thus, impair the ability of the host to recall vaccine-induced memory responses (Radwanska et al., 2008).

#### Protozoan parasites and vaccines against viral pathogens

Studies in mice suggested that *Plasmodium* infection is deleterious to pre-existing levels of heterologous antibodies. Specifically, *P*. *chabaudi* blood-stage infection of influenza-immune inbred and outbred mice resulted in a transient drop in influenza-specific antibodies and antibody secreting cells in the bone marrow (Ng et al., 2014). These results were confirmed by another study in which Malian children with pre-existing tetanus, measles, HBV vaccination had an accelerated decline in vaccine-specific IgG after acute malaria episodes (Banga et al., 2015) due to binding of *Plasmodium*-infected erythrocytes to bone marrow stromal cells that may disrupt the survival signals of long-lived plasma cells (Rogers et al., 2000; Kinyanjui et al., 2007; Banga et al., 2015). Additionally, BAFF receptor, which promotes survival of antibody secreting plasma cells, was found to be downregulated in splenic and bone marrow plasma cells, while circulating BAFF levels and apoptotic plasma cells increased in the bone marrow of *Plasmodium*-infected mice. These results were confirmed by the observation that *Plasmodium*-induced polyclonal B cell activation and elevated levels of immunoglobulins results in apoptosis of long-lived plasma cells through a CD32-dependent mechanism [reported in (Banga et al., 2015)]. Surprisingly, modest positive effects of pre-existing *Plasmodium* and helminths infections on vaccines efficacy of HPV vaccine was observed among young girls displaying *Plasmodium* parasitemia (Brown et al., 2014). Additionally, post-immunization measles antibody titers were significantly higher in the vaccinated children positive for *P*. *falciparum* infection than those without malaria parasites in the blood (Smedman et al., 1986).

*Leishmania major* is another protozoan that affects the viral vaccines-induced immune response. In BALB/c mice, infection with *L*. *major* reduced the CD8+ T cell-specific immune response induced by HIV-1 DNA vaccine suggesting that Th2 cell response caused by *L*. *major* infection can negatively affect vaccine efficacy (Robinson et al., 2004). Limited studies have been conducted to indicate contribution of Trypanosomes on viral vaccines. Foot-and-mouth disease virus vaccine was evaluated in cattle infected with *T*. *congolense* and despite significant depression in antibodies titers, their subsequent response to live virus challenge was not significantly different from the uninfected controls suggesting persistence of functional immunity (Sharpe et al., 1982). Antibody responses against classical swine fever virus vaccine were significantly decreased in *T*. *evansi*-infected pigs as compared to uninfected animals (Holland et al., 2003). Thus, *Trypanosoma* affects antibody responses against viral vaccine antigens but may or may not affect protection offered by the vaccines.

The Rotavirus vaccine efficacy was evaluated in diarrhea-associated co-infections in India, including those caused by *Cryptosporidium*. Vaccine efficacy decreased from 49.6 to 60.6% in the presence of co-infections (Praharaj et al., 2019). In another study, enteric co-infections including those by *Cryptosporidium and Giardia* did not affect the effectiveness of rotavirus vaccine. Therefore, lower vaccine effectiveness reported in low-income countries could not be explained only because of co-infection with *Cryptosporidium* (Mokomane et al., 2018).

Overall, it appears that the effect of protozoan infections on antibodies protection against vaccine antigens of different viruses is variable but the correlation of change in antibody levels with the failure or success of the vaccines in protection against the specific viral infection remains to be investigated more extensively.

## Concluding remarks

Mammalian hosts encounter protozoan and other pathogens that are either transmitted consecutively or simultaneously. The impact of immunosuppression by protozoan pathogens often increases the co-infecting bacterial burden in the affected organs, thus increasing the severity of diseases they cause. It would not be surprising if chronic protozoan infection and the resulting sustained immunosuppression can activate latent infection, such as by *M*. *tuberculosis* in malaria endemic regions. Therefore, efforts to manage, treat bacterial and protozoan infections or develop novel vaccines need to consider the presence of co-infections because that could have a dramatic influence on host susceptibility to disease and the design of treatment approaches. We document variable effects of protozoan infections on bacterial and viral vaccines with significant effects on reduction in efficacy of bacterial vaccines. Some viral vaccine response remains unaltered by the presence of infecting protozoan, while antibody titer is either increased or decreased in other cases albeit their impact on protection from infection remains unclear. Therefore, booster doses of vaccines may be needed when breakthrough infections start appearing in a particular geographic region, such as tetanus toxoid booster is often required under specific circumstances. In fact, vaccines are often recommended to protect from recurrence of viral diseases such as shingles in otherwise healthy and middle-aged adults who were infected with chickenpox as children. Furthermore, a more targeted treatment approach needs to be developed for specific co-infections to avoid toxicity due to excessive use of chemotherapeutics. For example, common metabolic pathways of co-infecting pathogens can maximize antimicrobials efficacy, reduce toxicity due to excessive drug use, and curb multidrug resistance emergence. Studies in Africa under World Health Organization examined antibody titer against measles in vaccinated infants with malaria parasite presence/absence. Extension of such studies in older children and adults in regions with chronic protozoan infection could reveal the full picture of their impact on viral vaccines and would lead to better understanding of vaccines efficacy, help document causes of breakthrough infections and employ approaches for improvement of protection by vaccines.

### Abbreviations and descriptions

**Bacille Calmette-Guerin (BCG)** is a vaccine against tuberculosis disease in regions where this disease is highly prevalent.

**Bacterial vaginosis (BV)** is caused by a localized inflammatory response against overgrowing natural microbiota of vagina.

**Enteroaggregative *E*. *coli* (EAEC)** is a pathogenic *E*. *coli* species that causes chronic diarrhea and is known because of its ability to form aggregates on intestinal mucosal surface.

**Cross-Reactive-Material-197 (CRM197)** is a mutant version of the diphtheria toxin rending a protein non-toxic.

**Diphtheria-tetanus-acellular pertussis (DTPa)** is a trivalent vaccine against three bacteria, *Corynebacterium diphtheriae*, *Clostridium tetani* and *Bordetella pertussis* given to children to prevent from serious diseases by these pathogens. Diphtheria causes breathing problem, tetanus results in tightening of muscles while pertussis is contagious disease that is also known as whooping cough.

**Heat shock protein 70 (HSP70)** is a universally expressed conserved protein in almost all living organisms that serves as chaperone for proteins for transport across membrane and also have been shown to function as potent stimulators of the innate immune system.

**Non-Typhoid Salmonella (NTS)** are group of Salmonella species that are not usually human pathogens unlike *Salmonella enterica* serovar Typhi that causes typhoid fever.

**Reactive oxygen species (ROS)** are oxygen containing oxygen containing reactive species produced during aerobic respiration. ROS produced by neutrophils and other phagocytes in mammalian hosts can kill organisms by causing permanent damage to DNA.

**Tuberculosis (TB)** is disease caused by respiratory bacterial pathogen, *Mycobacterium tuberculosis* that can be visualized under the microscope after acid-fast staining.

## Author contributions

LA and SR wrote the initial draft and prepared Table 1. NP prepared Figure 1. All authors edited and read the final version and approved it for submission.

## Funding

This work was supported by the National Institutes of Health (R01AI137425) grant to NP.

## Conflict of interest

The authors declare that the research was conducted in the absence of any commercial or financial relationships that could be construed as a potential conflict of interest.

## Publisher’s note

All claims expressed in this article are solely those of the authors and do not necessarily represent those of their affiliated organizations, or those of the publisher, the editors and the reviewers. Any product that may be evaluated in this article, or claim that may be made by its manufacturer, is not guaranteed or endorsed by the publisher.
